# *HOXB4* Gene Expression Is Regulated by CDX2 in Intestinal Epithelial Cells

**DOI:** 10.1371/journal.pone.0164555

**Published:** 2016-10-18

**Authors:** Steffen Jørgensen, Mehmet Coskun, Keld Mikkelsen Homburg, Ole B. V. Pedersen, Jesper T. Troelsen

**Affiliations:** 1 Department of Science and Environment, Roskilde University, Roskilde, Denmark; 2 Department of Clinical Immunology, Naestved Hospital, Naestved, Region Zealand, Denmark; 3 Department of Gastroenterology, Medical Section, Herlev Hospital, University of Copenhagen, Copenhagen, Denmark; University of Kansas School of Medicine, UNITED STATES

## Abstract

The mammalian *Caudal*-related homeobox transcription factor 2 (CDX2) plays a key role in the homeobox regulatory network and is essential in regulating the expression of several homeobox (*HOX)* genes during embryonic development, particularly in the gut. Genome-wide CDX2 chromatin immunoprecipitation analysis and expression data from Caco2 cells also suggests a role for CDX2 in the regulation of *HOXB4* gene expression in the intestinal epithelium. Thus, the aim of this study was to investigate whether *HOXB4* gene expression is regulated by CDX2 in the intestinal epithelium. We demonstrated binding of CDX2 to four different CDX2 binding sites in an enhancer region located upstream of the *HOXB4* transcription start site. Mutations in the CDX2 binding sites reduced *HOXB4* gene activity, and knock down of endogenous CDX2 expression by shRNA reduced *HOXB4* gene expression. This is the first report demonstrating the CDX2 regulation of HOXB4 gene expression in the developed intestinal epithelium, indicating a possible role for HOXB4 in intestinal homeostasis.

## Introduction

Homeobox (HOX) proteins are important regulators during development and the formation of the anterior and posterior body plane in vertebrates. The 39 different *HOX* genes in humans can be divided into four different clusters, *HOXA*, *HOXB*, *HOXC* and *HOXD*, which are located on different chromosomes[1]. Each cluster contains 9–11 *HOX* genes. *HOX* genes are transcription factors belonging to the HOX protein family that share a DNA-binding domain, termed the HOX domain. The HOX domain is comprised of 60 amino acids that bind DNA in a sequence-dependent manner[2]. In adults, *HOX* gene expression is organ-specific, and the deregulation of specific *HOX* genes has been associated with cancer development[3]; these genes include *HOXA9*, which has been implicated in the development of acute myeloid leukemia[4], *HOXD3*, which increases invasiveness and metastasis in lung cancer[5], and both *HOXA4* and *HOXD10*, which are overexpressed in colorectal cancer[6].

Another member of the HOX family is HOXB4, which is expressed in different tissues such as colon and liver[7] and is a possible marker in cervical cancer[8]. In hematopoietic stem cells, overexpression of HOXB4 increases proliferation, and expression is regulated by the transcription factor GATA-2[9]. However, despite the central role of HOXB4 in these processes, the regulation of *HOXB4* is still poorly understood.

The *Caudal*-related homeobox 2 (*CDX2*) gene is specifically expressed early after fertilization in the trophectoderm and is important for the correct formation of the blastocyst and subsequent implantation in the uterus[10]. During later development, the *CDX2* gene is specifically expressed posteriorly in the tailbud. The gene encoding the transcription factor CDX2 is also a member of the parahox gene family. DNA interactions mediated by the homeodomain of CDX2 can activate gene expression in a manner similar to the HOX proteins. CDX2 also plays an important role during development and establishment of the gastro-intestinal tract, and loss of CDX2 during development is fatal[11]. Later in life, CDX2 expression is limited to the intestinal epithelium and plays an important role in maintaining the crypt/villus axis and cellular differentiation along the axis[12]. Loss of Cdx2 expression in the mouse intestinal epithelium inhibits the development of enterocytes and is fatal, underscoring the importance of CDX2 in intestinal homeostasis[13]. Moreover, CDX2 acts as a tumor suppressor, and inhibition of CDX2 expression increases the invasiveness of colon cancer[14]. We conducted the study presented here to determine whether CDX2 is involved in the gene expression of *HOXB4*.

## Materials and Methods

### Cell lines

SW480 (ATCC: CCL-228), Caco2 (ATCC: HTB-37) and HEK293 (ATCC: CRL-1573) cells were cultured as monolayers in Dulbecco's Modified Eagle’s Medium (DMEM) supplemented with 10% heat inactivated fetal calf serum (FCS), 100 μg/ml streptomycin and 100 U/ml penicillin. All cell lines were cultured in a humidified atmosphere under 5% CO_2_.

### Viral transduction and CDX2 knockdown

An SW480 cell line with stable *CDX2* knock down was created using the lentiviral pLKO.1 puro shRNA expression vector. The shRNA expression vector (pLKO.1 puro) was a kind gift from Dr. Bob Weinberg (Plasmid #8453, Addgene, Cambridge, MA, USA)[15]. pLKO.1 puro was digested with *AgeI* and *EcoRI*, and the DNA oligos CDX2 shRNA 735 Forward and CDX2 shRNA 735 Reverse were hybridized and inserted into pLKO.1. A lentiviral control vector containing scrambled non-target shRNA (pLKO.1 scramble) was used as a negative control and was a kind gift from Dr. David Sabatini (Plasmid #1864, Addgene)[16]. Next, 6x10^6^ HEK293 cells were plated in 6-well plates one day prior to transfection. HEK293 were co-transfected with either scrambled shRNA or *CDX2* shRNA expressing lentivirus, psPAX, which was a gift from Malin Parmar (Plasmid #35002, Addgene), and pMD2.G, which was a gift from Didier Trono (Plasmid #12259, Addgene). The transfected HEK293 cells were maintained for 48 hours in DMEM supplemented with heat inactivated 10% FCS. The supernatant was then sterile filtered using a 0.45-μM filter and added to 5x10^5^ SW480 cells, followed by transduction for 24 hours. After 24 hours, the medium was replaced with DMEM supplemented with 10% heat inactivated FCS and 10 μg/ml puromycin (Life Technologies, Carlsbad, CA, USA), and the cells were reseeded twice per week for 3 weeks. Thereafter, cells were reseeded twice per week and cultured in DMEM supplemented with 10% heat inactivated FCS. *CDX2* knock down was verified by RT-qPCR and western blotting.

### *HOXB4* promoter/enhancer cloning

A potential CDX2 binding region in the *HOXB4* enhancer was identified using previously published chromatin immunoprecipitation-sequencing (ChIP-seq) data [17], which was uploaded to the UCSC Genome Browser (version hg18).

The BAC clone RP11-111C6, which covers chromosome 17, positions ‒48583316 to ‒ 48578320 (GENBANK ID: NC_000017.11), was obtained from the BACPAC Resource Centre (Oakland, CA, USA) and used as a template to amplify the *HOXB4* promoter and enhancer region. The *HOXB4* promoter and enhancer region was cloned using an In-Fusion^tm^ cloning kit (Clontech, Saint-Germain-en-Laye, France). A 4997-bp fragment was amplified using Phusion HOTSTART II HD polymerase (Thermo Fisher Scientific, Waltham, MA, USA), the Infusion HOXB4 Enhancer/promoter forward primer and the Infusion HOXB4 reverse primer (Table 1). The pGL4.10 luciferase reporter plasmid (Promega, Nacka, Sweden) was digested with *HindIII* according to the manufacturer’s protocol (Thermo Fisher Scientific), and the promoter and enhancer were inserted using the In-Fusion^tm^ HD cloning kit (Clontech) according to the manufacturer’s protocol (Clontech). A 2850-bp fragment excluding the up-stream CDX2 binding sites was amplified using Phusion HOTSTART II HD polymerase (Thermo Fisher Scientific), the Infusion HOXB4 promoter forward primer and the Infusion HOXB4 promoter reverse primer (Table 1). The pGL4.10 luciferase reporter plasmid (Promega) was digested with *HindIII* according to the manufacturer’s protocol (Fisher Scientific), and the *HOXB4* promoter was inserted using the In-Fusion^tm^ HD cloning kit (Clontech) according to the manufacturer’s protocol (Clontech)

**Table 1:** DNA oligo sequences used in this study.

Name	Sequence
CDX2 shRNA 735 Forward	5’- CGGCAAATATCGAGTGGTGTACACCTCGAGGTGTACACCACTCGATATTTGTTTTTG-‘3
CDX2 shRNA 735 Reverse	5’- AATTCAAAAACAAATATCGAGTGGTGTACACCTCGAGGTGTACACCACTCGATATTTG-‘3
Infusion HOXB4 Promoter Reverse Primer	5’-CCGGATTGCCAAGCTGGTGGTGTAATAAAAGTCCTTTTG-‘3
Infusion HOXB4 Enhancer/Promoter Forward Primer	5'-CTCGGCGGCCAAGCTATTGATGAGAACGATTCTTCGG-’3
Infusion HOXB4 Enhancer/Promoter Reverse Primer	5'-CCGGATTGCCAAGCTTAATTTCTGGGAATTGCCCACAAA-‘3.
Infusion HOXB4 Move Forward	5'-CTAGGCGAGTTCATA TATGTGTTTAGAAGGAAAGGAC-'3
Infusion HOXB4 Move Reverse	5'-GTCTGCCTGACTGCCTGTGGTCAGATCGATTGGC-'3
EMSA-CDX2-Site-1 Forward	5'-TCAGGCCTTTCTGGGTGCCT-'3
EMSA-CDX2-Site-1 Reverse	5'-AAGGCACCCAGAAAGGCCTG-'3
EMSA-CDX2-Site-2 Forward	5'-GGTGGTGTAATAAAAGTCCT-'3
EMSA-CDX2-Site-2 Reverse	5'-AAGGACTTTTATTACACCAC-'3
EMSA-CDX2-Site-3 Forward	5'-GGATATTTAATGACGGGCAA-'3
EMSA-CDX2-Site-3 Reverse	5'-ATTGCCCGTCATTAAATATC-'3
EMSA-CDX2-Site-4 Forward	5'-TGAGGAACCATAAAAGATGC-'3
EMSA-CDX2-Site-4 Reverse	5'-TGCATCTTTTATGGTTCCTC-'3
EMSA Sucrase-isomaltase probe Forward	5’-ACTGACTTTATTAACTTTGTGACC-’3
EMSA Sucrase-isomaltase probe Forward	5’-AGGTCACAAAGTTAATAAAGTCAG-’3
EMSA unspecific probe Forward	5’-AACGTAGCTGATCGAATCGGTTAC-’3
EMSA unspecific probe Reverse	5’-AGTAACCGATTCGATCAGCTACGT-’3
HOXB4 ChIP PCR Forward	5'-GCCAGATGATATAGGGCCCC-'3
HOXB4 ChIP PCR Reverse	5'-AAGGTGGGGAAATGGGGTTC-'3
HOXB4 qPCR Forward	5'-CTGGATGCGCAAAGTTCAC
HOXB4 qPCR Reverse	5'-AGCGGTTGTAGTGAAATTCCTT-'3
CDX2 qPCR Forward	5'-ACTACAGTCGCTACATCACCA-'3
CDX2 qPCR Reverse	5'-GAAGACACCGGACTCAAGGG-'3
RPLP0 qPCR Forward	5'-GCAATGTTGCCAGTGTCTG-'3
RPLP0 qPCR Reverse	5'-GCCTTGACCTTTTCAGCAA-'3
HOXB4 CDX2 ChIP PCR Forward	5'-GCCAGATGATATAGGGCCCC-'3
HOXB4 CDX2 ChIP PCR Reverse	5'-AAGGTGGGGAAATGGGGTTC-'3

### CDX2 binding site mutations

Four different constructs containing the *HOXB4* promoter/enhancer region (4997-bp fragment) and in which each of the CDX2 binding sites, sequence a/cATAAAa/t[18], were replaced with the *EcoRI* cleavage sequence, GAATTCG, were synthesized by Eurofins (Eurofins MWG Operon, Ebersberg, Germany). The HOXB4 pGL4.10 reporter plasmid was digested with *NdeI* (Thermo Fisher Scientific) and *PsyI* (Thermo Scientific) according to the manufacturer’s protocol. Each of the four CDX2 mutated constructs was used as template, and a 1724-bp fragment containing the mutated CDX2 binding site was amplified using Phusion HotStart II polymerase (Thermo Fisher Scientific), following the manufacturer’s protocol, and the Infusion HOXB4 Move forward and reverse primers (Table 1). The fragments were inserted in the *NdeI*- and *PsyI*-digested *HOXB4* pGL4.10 reporter plasmid using the In-Fusion^tm^ HD cloning kit (Clontech) according to the manufacturer’s instructions, resulting in four different plasmids with each of the CDX2 binding sites mutated.

### Reporter gene assays

One day prior to transfection, SW480 cells were seeded in 24-well plates at a density of 5x10^4^ cells/well and transiently transfected the following day. The cells were transfected using polyethylenimine at a final concentration of 2 μM and 0.3 μg DNA/well. Twenty-five nanograms of luciferase reporter gene plasmid and 50 ng of β-galactosidase expression plasmid were used per well, and the DNA concentration was adjusted to 0.3 μg with pBluescript SK+ (Stratagene, La Jolla, CA, USA). Forty-eight hours post-transfection, cells were harvested and lysed; luciferase and β-galactosidase activities were determined using the Dual Light^tm^ luminescent reporter gene assay (Applied Biosystems, Naerum, Denmark), and luminescence was determined using a *GloMax*® 96 Microplate Luminometer (Promega) according to the manufacturer’s instructions.

### Protein extraction and immunoblotting

Total proteins were extracted from the lentiviral-modified shRNA-expressing SW480 cells (scrambled shRNA or *CDX2* shRNA) using Trizol (Life Technologies) as described by the manufacturer. Ten micrograms of total protein were analyzed by SDS-PAGE using a NuPAGE 4–12% Bis-Tris protein gel (Life Technologies) followed by transfer to a PVDF membrane via electroblotting using the iBlot Western blotting system (Life Technologies). The membrane was incubated with primary antibodies against human CDX2 (1:300, mouse monoclonal, BioGenex Laboratories, Fremont, CA) or glyceraldehyde 3-phosphate dehydrogenase (GAPDH, 1:300, mouse monoclonal, Millipore, Darmstad, Germany).

### Electrophoretic mobility shift assay

The amplified region of the *HOXB4* promoter and enhancer was analyzed using the JASPAR database with a relative profile score threshold setting of 80% [19]. Four different probe sets were designed to cover CDX2 binding sites in the *HOXB4* enhancer at position ‒2662 to ‒2672 (site-4) on the minus strand, position ‒2737 to ‒2747 (site-3) on the plus strand, position ‒2836 to ‒2846 (site-2) on the minus strand, and position ‒3030 to ‒3040 (site-1) on the plus strand. The electrophoretic mobility shift assay (EMSA) was performed as previously described[20]. Briefly, nuclear extracts from confluent Caco2 cells were incubated with p32-labeled probes for site-1, -2, -3, or -4. A competition assay was performed using either an unlabeled sucrase isomaltase probe containing a known CDX2 binding site (positive control) or unlabeled HOXB4 probes corresponding to each of the four identified potential CDX2 binding sites (site-1, -2, -3-, or -4). A supershift assay was performed using a human CDX2 antibody (BioGenex) or influenza hemagglutinin (HA) as a negative control (described below).

### RNA extraction and RT-qPCR

Total RNA was extracted from SW480 cells using an E.Z.N.A. Total RNA KIT I (OMEGA BIOTEK, Norcross, GA). RNA extraction was performed according to the manufacturer’s instructions. cDNA was synthesized according to the manufacturer’s instructions, using qSCRIPT cDNA Supermix (Quanta Biosciences, Gaithersburg, MD). Relative quantification was calculated using the ΔΔC_T_ method, and ribosomal protein large P0 (RPLP0) was used as a reference gene[21].

### Chromatin immunoprecipitation

Confluent Caco2 cells were cross-linked, sonicated, and immunoprecipitated with an antibody specific for either human CDX2 (BioGenex) or an antibody specific for the influenza hemagglutinin (HA) epitope (rabbit polyclonal α-HA; Santa Cruz Biotechnology Inc., Heidelberg, Germany) as a negative control, as previously described[22]. Purified immunoprecipitated DNA was compared to input DNA, which corresponded to non-immunoprecipitated sheared cross-linked chromatin, via qPCR analysis. The primers used to amplify the human genomic sequences of *HOXB4* are listed in Table 1, and quantification of the chromatin immunoprecipitation (ChIP)-DNA was performed using qPCR in triplicate with a Stratagene MX3002 (Stratagene) and a QuantiTect SYBR Green PCR Kit (Qiagen, Duesseldorf, Germany). Enrichment was calculated as described elsewhere[23].

### Statistical analysis

Statistical analysis was performed using unpaired Student’s *t*-tests, and values are shown as the mean and standard deviation (SD). P-values of less than 0.05 were considered significant.

## Results

### CDX2 binds to the *HOXB4* enhancer

**Fig 1:**
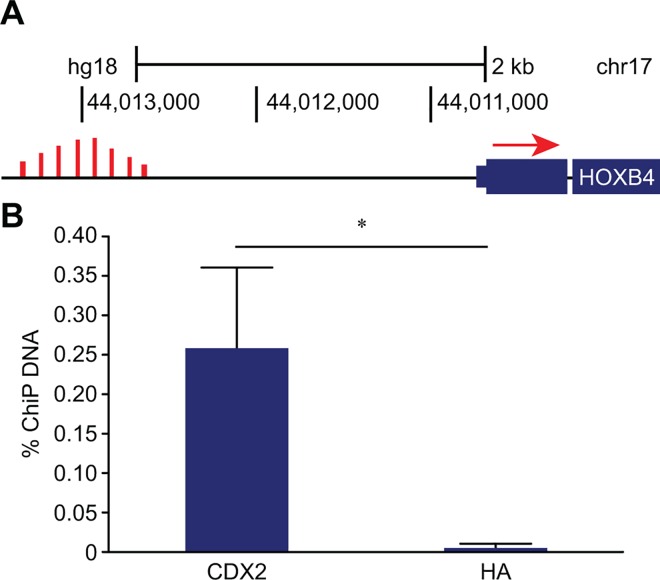
Illustration of the CDX2 binding region in the *HOXB4* enhancer region and analysis of CDX2 interactions with the *HOXB4* enhancer region. Fig 1A) Genome browser CDX2 ChIP-seq peaks from a dataset generated by Boyd et al.[17]. Fig 1B) ChIP-PCR with primers covering the CDX2 binding region within the *HOXB4* enhancer. Equal amounts of input DNA were used for CDX2 ChIP-PCR. Hemagglutinin (HA) was used as a negative control. The mean (n = 4) and SD are shown. *P< 0.05.

Genome-wide CDX2 ChIP-seq data from a published report[17] on the intestinal cell line Caco2 revealed a potential CDX2 binding region in the *HOXB4* enhancer, located from 2640 to 3050 bp upstream of the *HOXB4* transcription start site (Fig 1A). Thus, to verify whether CDX2 binds to this region, we performed qPCR on chromatin obtained from Caco2 cells immunoprecipitated with an anti-CDX2 or an anti-HA (negative control) antibody. In ChIP assays, chromatin obtained from anti-CDX2 antibody immunoprecipitates showed significant enrichment of the *HOXB4* enhancer (49-fold, *P*<0.05) compared to the negative control (Fig 1B).

### *HOXB4* expression is modulated by CDX2

**Fig 2:**
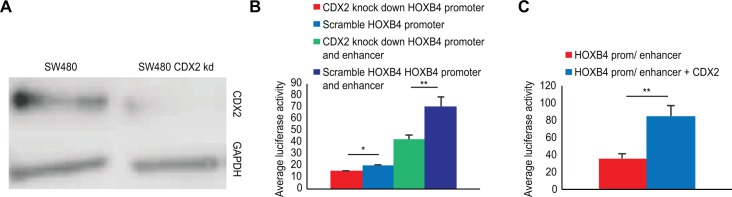
The effects of *CDX2* knock down and overexpression on HOXB4 expression in the SW480 cell line and functional analysis of 4 different CDX2 binding sites in the *HOXB4* enhancer region. Fig 2A) Western blot analysis of CDX2 expression levels in SW480 cells treated with scrambled shRNA and cells in which *CDX2* was knocked down. GADPH was used as a control to analyze equal amounts of total cellular protein. Fig 2B) The effects of *CDX2* knock down on the promoter activities of reporter constructs containing the *HOXB4* promoter alone or the *HOXB4* promoter including the enhancer region. Fig 2C) Transient transfection of the *HOXB4* promoter and enhancer reporter construct in SW480 cells with and without co-transfection of a CDX2 expression plasmid. The mean (n = 4) and SD are shown. *P<0.05 and **P<0.01

To determine whether endogenous *HOXB4* expression is regulated by CDX2, we performed stable shRNA-mediated knock down of endogenous *CDX2* in SW480 cells. This cell line was chosen because we were unable to generate a Caco2 cell line with stable *CDX2* knock down, a limitation that has also been reported by other groups[18]. In SW480 cells, endogenous CDX2 expression was effectively knocked down (Fig 2A). Moreover, both *CDX2* and *HOXB4* mRNA levels were decreased in SW480^*CDX2*^ compared to SW480^NEG^ cells (data not shown), indicating that CDX2 is involved in maintaining the endogenous expression of *HOXB4* in SW480 cells.

To verify the possible role of CDX2 in the regulation of *HOXB4* expression, a luciferase reporter construct containing the human *HOXB4* enhancer and promoter as well as another construct containing only the *HOXB4* promoter were created. The activity of the reporter constructs containing the *HOXB4* promoter or *HOXB4* promoter and enhancer were measured both in SW480^*CDX2*^ and SW480^NEG^ cells. The *HOXB4* promoter exhibited activity on its own when comparing the expression levels in SW480^*CDX2*^ and SW480^NEG^ cells (Fig 2B). The activity of the luciferase construct containing both the *HOXB4* enhancer and promoter was significantly reduced (*P*<0.01) when examining the expression levels in SW480^*CDX2*^ compared to SW480^NEG^ cells (Fig 2B). This result indicates that CDX2 is involved in the transcriptional regulation of *HOXB4*.

To further investigate the role of CDX2 in *HOXB4* expression, we performed a co-transfection experiment in SW480 cells. The *HOXB4* enhancer and promoter reporter construct was transiently transfected into SW480 cells with or without the CDX2 expression plasmid. When overexpressing CDX2, luciferase activity driven by the *HOXB4* enhancer and promoter construct was significantly higher (2.4-fold, *P*<0.01) relative to cells without co-transfection of the CDX2 expression vector (Fig 2C).

### CDX2 interacts with the *HOXB4* enhancer region

**Fig 3:**
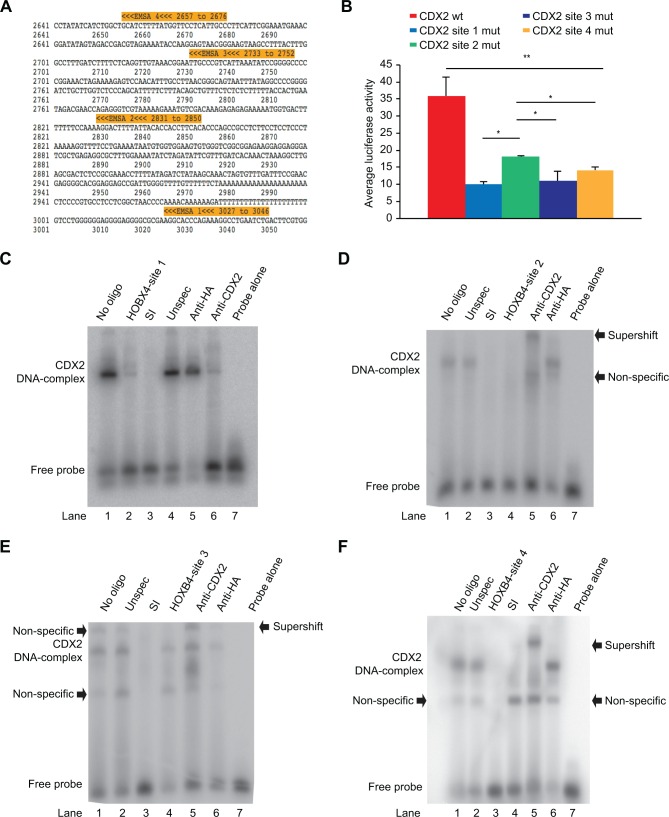
Functional and EMSA analyses of CDX2 binding sites in the *HOXB4* enhancer region. Fig 3A) Positions of the four potential CDX2 binding sites and the EMSA probes covering these sites. Fig 3B) Reporter gene assays with four different luciferase constructs containing the *HOXB4* enhancer with wild-type CDX2 binding sites or four distinct reporter constructs containing individual mutations in the four CDX2 binding sites (*HOXB4* site-1, -2, -3 or 4). The mean (n = 4) and SD are shown. *P<0.05 and **P<0.01. Fig 3C–3F) EMSAs with probes for the four different CDX2 binding sites (HOXB4 site-1, -2, -3 and 4). One hundred-fold unlabeled probe was added to the competition reactions as well as sucrase isomaltase (SI) or unspecific probe (unspec). Influenza hemagglutinin (HA) antibody (negative control) or CDX2 antibody (CDX2) was used in the supershift assay. Non-specific bands and supershifts indicated by black arrows.

We used computational DNA sequence analysis to identify potential CDX2 binding sites in the *HOXB4* enhancer region. This analysis revealed four predicted and potential CDX2 binding sites within the *HOXB4* enhancer region (Fig 3A). To further analyze the function of each of the four CDX2 binding sites, mutations were introduced into each CDX2 binding site (*HOXB4* site-1, -2, -3, and -4). The enhancer activity of each construct was measured using transient transfection in SW480 cells, and mutation of all four sites individually significantly reduced *HOXB4* enhancer and promoter luciferase activity (*P*<0.01 for all) compared to the wild type reporter construct (Fig 3B). When comparing the reduction in enhancer activity between the four CDX2 binding sites, mutation of *HOBX4* site 2 reduced the activity to a lesser extent compared to *HOXB4* site-1, -3 and -4. (*P*<0.05)

In addition to the functional analysis of the *HOXB4* enhancer/promoter constructs containing mutations within the CDX2 binding sites (*HOXB4* site-1, -2, -3 or -4), we performed EMSA with oligonucleotides covering each of the four potential CDX2 binding sites and nuclear extracts from Caco2 cells (Fig 3C–3F). The data revealed that all four probes were able to induce the formation of a CDX2/DNA complex (Fig 3C–3F, lane 1). However, two non-specific complexes for site-3 and one non-specific complex for site-4 were revealed (Fig 3E and 3F, lane 1, respectively). The binding specificity of these complexes was examined in competition assays in which excess unlabeled cold probes, specifically a sucrose isomaltase probe, a probe containing the CDX2 consensus sequence, or probes containing CDX2 binding site sequences (*HOXB4* site-1, -2, -3 or -4), or an unspecific oligonucleotide were utilized. Unlabeled *HOXB4* site-2 and site-4 probes completely out-competed CDX2/DNA complexes (Fig 3C, lane 4, and Fig 3E, lane 3). However, cold *HOXB4* site-1 and site-3 probes only partially out-competed the CDX2/DNA complex (Fig 3B, lane 2, and Fig 3D, lane 4). In contrast, the formation of the CDX2/DNA complex was completely eliminated in all EMSAs by competition with the unlabeled sucrase isomaltase probe (Fig 3B–3D, lane 3, and Fig 3E, lane 4), whereas the unspecific probe did not affect CDX2/DNA complex formation (Fig 3C, lane 4, and Fig 3D and 3E, lane 2).

To verify that CDX2 is present in the CDX2/DNA complex, supershift assays were performed with anti-CDX2 and anti-HA (negative control) antibodies. The addition of an antibody against CDX2 effectively supershifted the CDX2/DNA complex in site-2, -3, and -4 (Fig 3C–3E, lane 5) but failed to supershift the CDX2/DNA complex in site-1 (Fig 3B, lane 6), although a partial reduction in the band intensity of the CDX/DNA complex was observed. Moreover, as expected, the CDX2/DNA complex did not supershift with the addition of an antibody against HA (Fig 3C, lane 5, and Fig 3D and 3E, lane 6).

## Discussion

The regulation of *HOXB4* expression is poorly understood in the intestinal epithelium and, until now, no other transcription factors in the intestinal epithelium have been shown to directly induce *HOXB4* expression. Here, we demonstrate that CDX2 regulates *HOXB4* gene expression in the intestinal epithelium-derived cell line SW480. Induction of *HOXB4* expression has previously been studied during fetal development, and expression is induced in the hindbrain by retinoic acid. The induction of HOXB4 expression is mediated by the retinoic acid response element located within the *HOXB4* enhancer[24]. *HOXB4* is also expressed during hematopoiesis and regulated by the transcription factor GATA-2 in hematopoietic stem cells and progenitor cells[9].

A reduction in *HOXB4* expression levels, achieved using siRNA-mediated *CDX2* knock down in the COLO320 cell line, has previously been demonstrated[25]. In the present study, we made similar observations in two different cell lines, Caco2 and SW480, both derived from colorectal cancers. However, Salari et al. did not investigate the possible CDX2-mediated induction of HOXB4. We demonstrate CDX2 binding to the HOXB4 enhancer region by ChIP-PCR and we have identified four CDX2 binding sites and performed a functional analysis of each site. Mutating each of the 4 sites significantly reduces enhancer activity compared to controls, but not equally. Mutating the HOXB4 site-2 affects promoter activity to a lesser extent compared to the mutation of HOXB4 site-1, HOXB4 site-3 and HOXB4 site-4.

Previous studies of CDX1, 2 and 4 have shown that they bind to the promoter of HOXA5, and it has been suggested that CDX4 modulates HOXA5 expression during embryonic development[26]. CDX2 expression along the crypt-villus axis varies, with the lowest CDX2 expression at the bottom of the crypt and increased expression during cellular differentiation[27]. The expression of HOXA5 also varies and increases during differentiation[28], and it is possible that the expression is mediated by CDX2. HOXA5 has been suggested to be a potential tumor suppressor in breast cancer[29]. CDX has also been shown to regulate HOXB8 expression during embryonic development. Furthermore, *in vitro* binding of CDX1, CDX2 and CDX4 has been described, and binding has been demonstrated by all three CDX transcription factors *in vivo*[30].

HOXB4 binding sites have been investigated in hematopoietic stem cells and progenitor cells, and HOXB4 has been shown to both up- and down-regulate the expression of target genes[31]. However, the binding of HOXB4 is dependent not only on HOXB4 but also on several different co-factors. These co-factors form complexes with HOB4 and are members of the TALE family, including MEIS, PBX and PREP, which play important roles in target sequence selection and binding[32]. However, the intestinal expression levels of these co-factors remain unclear and must be investigated to identify HOXB4 target genes in the intestinal epithelium.

The function of the CDX2-mediated induction of HOXB4 in intestinal differentiation and homeostasis is not known. As *HOX* genes are transcription factors, their downstream targets must be identified to clarify the function of HOXB4 expression in the intestinal epithelium. It is possible that HOXB4 induction plays a role in cellular differentiation or intestinal homeostasis.

We show that CDX2 binds to the HOXB4 enhancer, regulates HOXB4 expression in intestinal-derived epithelial cell lines, and has a possible role in the regulation of HOXB4 expression in the intestinal epithelium and intestinal homeostasis.
